# Naturally occurring low sociality in female rhesus monkeys: A tractable model for autism or not?

**DOI:** 10.1186/s13229-024-00588-3

**Published:** 2024-01-31

**Authors:** Ozge Oztan, Laura A. Del Rosso, Sierra M. Simmons, Duyen K. K. Nguyen, Catherine F. Talbot, John P. Capitanio, Joseph P. Garner, Karen J. Parker

**Affiliations:** 1 Department of Psychiatry and Behavioral Sciences, Stanford University, 1201 Welch Rd., MSLS P-104, Stanford, CA 94305 USA; 2 Department of Comparative Medicine, Stanford University, 300 Pasteur Dr., Edwards R348, Stanford, CA 94305 USA; 3 California National Primate Research Center, 1 Shields Ave., Davis, CA 95616 USA; 4 School of Psychology, Florida Institute of Technology, 150 W. University Blvd., Melbourne, FL 32901 USA; 5 Department of Psychology, University of California, 1 Shields Ave., Davis, 95616 USA

**Keywords:** Animal model, Arginine vasopressin, Autism spectrum disorder, Cerebrospinal fluid, Dominance rank, Female, Oxytocin, Rhesus macaque, Social functioning, Social responsiveness scale

## Abstract

**Background:**

Autism spectrum disorder (ASD) is characterized by persistent social interaction impairments and is male-biased in prevalence. We have established naturally occurring low sociality in male rhesus monkeys as a model for the social features of ASD. Low-social male monkeys exhibit reduced social interactions and increased autistic-like trait burden, with both measures highly correlated and strongly linked to low cerebrospinal fluid (CSF) arginine vasopressin (AVP) concentration. Little is known, however, about the behavioral and neurochemical profiles of female rhesus monkeys, and whether low sociality in females is a tractable model for ASD.

**Methods:**

Social behavior assessments (ethological observations; a reverse-translated autistic trait measurement scale, the macaque Social Responsiveness Scale-Revised [mSRS-R]) were completed on *N* = 88 outdoor-housed female rhesus monkeys during the non-breeding season. CSF and blood samples were collected from a subset of *N* = 16 monkeys across the frequency distribution of non-social behavior, and AVP and oxytocin (OXT) concentrations were quantified. Data were analyzed using general linear models.

**Results:**

Non-social behavior frequency and mSRS-R scores were continuously distributed across the general female monkey population, as previously found for male monkeys. However, dominance rank significantly predicted mSRS-R scores in females, with higher-ranking individuals showing fewer autistic-like traits, a relationship not previously observed in males from this colony. Females differed from males in several other respects: Social behavior frequencies were unrelated to mSRS-R scores, and AVP concentration was unrelated to any social behavior measure. Blood and CSF concentrations of AVP were positively correlated in females; no significant relationship involving any OXT measure was found.

**Limitations:**

This study sample was small, and did not consider genetic, environmental, or other neurochemical measures that may be related to female mSRS-R scores.

**Conclusions:**

Dominance rank is the most significant predictor of autistic-like traits in female rhesus monkeys, and CSF neuropeptide concentrations are unrelated to measures of female social functioning (in contrast to prior CSF AVP findings in male rhesus monkeys and male and female autistic children). Although preliminary, this evidence suggests that the strong matrilineal organization of this species may limit the usefulness of low sociality in female rhesus monkeys as a tractable model for ASD.

## Background

Autism spectrum disorder (ASD), like most neurodevelopmental and neuropsychiatric conditions, is currently diagnosed on the basis of behavioral criteria (i.e., persistent social interaction impairments; the presence of restricted and repetitive patterns of behavior) [1]. ASD lacks a robust laboratory-based diagnostic test and effective medications for its core features because its underlying biology remains poorly understood. One reason for these gaps in knowledge is that there has been an overreliance on animal models that fundamentally lack the complex cognitive and social skills required to model ASD’s core behavioral features. Consequently, we [2] and others [3, 4] have argued that primate models, which have greater behavioral and biological homology to humans, are more likely to yield streamlined translation and clinical impact for ASD.

Accordingly, we have used naturally occurring low sociality in male rhesus monkeys to model and study the social interaction impairments of ASD [5], focusing on behavioral phenotyping, biomarker discovery, and pharmacological testing. This work has determined that low-social males initiate fewer social interactions [6, 7], show abnormalities in species-typical perception of, and responses to, social stimuli [8, 9], exhibit a greater burden of autistic-like traits [10], and incur more traumatic injuries [11] than their socially competent male peers. Naturally occurring low sociality is also stable within individual males across time [7], unrelated to dominance rank [7, 9, 10, 12], and highly heritable [12]. In a targeted biomarker discovery study, we also found that cerebrospinal fluid (CSF) concentration of the ‘social’ neuropeptide arginine vasopressin (AVP) [13–16] is a robust classifier of low versus high sociality, and that it predicts quantitative social and autistic-like trait variation [as measured by the macaque Social Responsiveness Scale-Revised (mSRS-R)] in males from the general rhesus monkey population. As one would expect of a biomarker, CSF AVP concentration exhibits stable within-individual consistency across multiple measurements in males [2, 17].

We next tested the translational utility of our monkey model, by measuring CSF AVP concentrations in several cohorts of children with and without ASD, a majority of which were male. We found that CSF AVP concentration identified male and female children with ASD accurately, and that boys with the lowest CSF AVP concentrations had the greatest clinical symptom severity [2, 18]. We also found that neonatal CSF AVP concentration was a significant predictor of a subsequent ASD diagnosis later in childhood, indicating that CSF AVP concentration was already low before the period when ASD first manifests [19]. Finally, we recently reported in a pilot phase IIa clinical trial that intranasal AVP treatment improved social abilities in children with ASD [20], again, in a sample that was mostly (83%) male.

We selected male rhesus monkeys as subjects in our initial studies due to ASD’s sex-biased prevalence (ASD impacts approximately four males for every one female) [21]. Yet, ASD does affect females. Females of many species have historically been excluded from research studies for a variety of reasons, including sex discrimination, concerns about hormonal variation and reproductive health, and budgetary constraints [22, 23]. However, exclusion of females from research studies can jeopardize identification of disease mechanisms and lead to sex-specific health disparities [24]. There is now a growing awareness of the importance of sex- and gender-inclusive research to ensure a more comprehensive and accurate understanding of biological processes and responses to interventions [25].

The goal of the present study, therefore, was to test whether our model of naturally occurring low sociality could be extended to female rhesus monkeys in our colony, as a prerequisite for developing a similar translational research program focused on low-social female monkeys and girls with ASD. Females with ASD face a higher risk of misdiagnosis, delayed diagnosis, or failure to be diagnosed. This is likely due to sex differences in the clinical manifestation of ASD symptoms, and reliance on diagnostic tools primarily standardized in males [26–31]. Girls are also more likely to compensate for their social interaction difficulties by “camouflaging” [32–34], a human psychological construct unlikely to be present in female monkeys, thereby enabling investigation of the biological underpinnings of manifest social impairment in females. However, female rhesus monkeys have substantially different life histories from male rhesus monkeys, as well as from female humans, with female rhesus monkeys maintaining a strong matrilineal social system in which females born into a group remain in that group for their entire lives and interact predominantly with female kin. Males, by contrast, typically emigrate from the natal troop at puberty. The dominance structure in rhesus macaques (within a group, males and females have separate dominance hierarchies) has been described as “despotic” instead of “egalitarian,” with despotic systems involving strict priority of access based on social power. An important and notable sex difference in this species is that for females in a social group, entire matrilines can be ranked against each other, with individuals within each matriline showing individual ranks of their own that are near each other. Males must earn their rank through aggression and formation of coalitions as they enter new groups; females are largely born into their rank [35–37]. Although we have not previously found dominance rank to be related to low sociality or autistic-like trait burden in male rhesus monkeys [7, 9, 10, 12], we evaluated it here given its centrality to the lives of female rhesus monkeys, and its potential to compromise model development. We also consider a role for oxytocin (OXT) in addition to AVP, given its well-known role in female social functioning [38–40]. Below we describe the first investigation of the interrelationships between social and non-social behavior frequencies, autistic-like trait burden, and neurochemical profiles of female rhesus monkeys, to evaluate their suitability as a tractable model for ASD.

## Methods

### Study design

Social behavior assessments detailed below were completed over a 6-month period on *N* = 88 female rhesus monkeys during the non-breeding season. (Restricting behavioral observations to the non-breeding season minimizes the potential impact of seasonal changes in macaque social behavior). Behavioral scores were tabulated, and then CSF and blood samples were collected from a subset of *n* = 16 monkeys across the frequency distribution of non-social behavior. Experimenters were blinded to monkeys’ behavioral data during biological sample collection and subsequent quantification of AVP and OXT concentrations.

### Subjects and study site

Subjects were *N* = 88 female rhesus monkeys (*Macaca mulatta*), born and reared at the California National Primate Research Center (CNPRC). All subjects lived in mixed age and sex groups. Groups contained an average of 142 individuals and ranged in size from 87 to 191 individuals per group. Each group was housed in a large, outdoor, half-acre field corral (30.5 m wide × 61 m deep × 9 m high). Subjects were housed among 11 of the 24 field corrals.

Subjects were tattooed and dye-marked periodically to facilitate easy identification for husbandry- and research-related procedures. Monkeys had ad libitum access to Lixit-dispensed water. Primate laboratory chow was provided twice daily, and fruit and vegetable supplements were provided weekly. Various toys, swinging perches, and other forms of enrichment in each corral, along with outdoor and social housing, provided a stimulating environment.

Subjects were an average of 2.10 years old and ranged in age from 1.72 to 2.46 years at the time of study enrollment. Eligibility criteria included: female, 1.6–2.6 years of age (i.e., pre-reproductive), socially housed in any of the outdoor field corrals (i.e., not housed indoors in individual cages), medically healthy, and not simultaneously enrolled in another CNPRC project. Pre-reproductive females were selected for three reasons: (1) we wanted to avoid collecting biological samples from pregnant females for animal welfare reasons; (2) pregnancy, lactation, and parental care substantially affect female rhesus monkey social behavior; and (3) our translational ASD research program is focused on early detection and intervention.

Dominance rank was assessed in each corral using an established CNPRC colony-wide protocol by behavioral management personnel who recorded aggressive and submissive interactions following food provisioning. Because each corral contained a different number of females, using the raw dominance rank was ineffective as a direct measure that could be compared across all subjects. Thus, dominance rank was calculated as the proportion of females in the group that the focal individual outranked, such that the highest-ranked individual had a value of 1 and the lowest-ranked individual had a value of 0 [41]. Dominance ranks are assessed monthly at CNPRC; thus, we used monkeys’ dominance ranks collected contemporaneously with their mSRS ratings for the purpose of statistical analysis.

### Quantitative social behavior observations

Subjects were observed unobtrusively in their home field corrals. Prior to conducting behavioral observations, observers became reliable on data collection with > 90% agreement [number of agreements divided by the (number of agreements + number of disagreements)] on all behavioral categories. Each observer conducted 10-min focal samples on subjects during two observation periods per day (0830–1030 and 1045–1300), four days per week, for two weeks, resulting in a total of 16 focal samples over a period of 160 min for 320 data points per subject. Each observer watched a maximum of nine subjects, residing in one to three corrals, during each two-week observation period. We used instantaneous sampling [42] in which we recorded, at 30-s intervals, whether the subject was engaged in any of the following behaviors: alone (subject is not within an arm’s reach of any other animal and is not engaged in play), proximity (subject is within an arm’s reach of another animal), contact (subject is touching another animal in a nonaggressive manner), groom (subject is engaged in a dyadic interaction with one animal inspecting the fur of another animal using its hands and mouth), or play (subject is involved in chasing, wrestling, slapping, shoving, grabbing, or biting accompanied by a play face [wide eyes and open mouth, without bared teeth] and/or a loose, exaggerated posture and gait; the behavior must have been deemed unaggressive to be scored) [2]. Behavioral data were scored such that an individual could be engaged in more than one behavioral category at the same time (e.g., playing while in proximity to another individual). Additionally, for all social behaviors, it was noted if the subject was interacting with its mother. After data collection was completed, the total frequency of each measure was summarized across all the behavior samples collected for each subject.

### mSRS-R rating instrument

At the conclusion of each two-week behavioral observation period (at least 1 h after the final observation was concluded and no more than 24 h after the last observation), observers rated each subject’s quantitative autistic-like trait burden using the 36-item mSRS [43], which had been reverse-translated from the social responsiveness scale, an instrument used in humans to assess autistic traits and to screen for ASD [44, 45]. Our rating instrument had been modified to employ a seven-point Likert scale (1 = total absence of the trait, 7 = extreme manifestation of the trait) for each item. Since only 17 of the original 36 mSRS items exhibit inter-rater and test-rest reliability as determined for both male and female rhesus monkeys [10], here we extracted and tabulated ratings for the 17 reliable items, which form the basis of the mSRS-R [10]. Prior to final summary, questions written in the infrequent direction were reverse scored such that higher scores always indicated greater impairment. Final summed mSRS-R total scores can range between 17 and 119.

### Subject selection for biological sampling

Monkeys were rank ordered on the total frequency they had been observed in non-social behavior, which is a reliable, high-throughput method for identifying low-social monkeys [2]. Non-social behavior frequency ranged from 30.9% to 79.4% of observations, with mean = 57.3%, standard deviation (SD) = 9.4%. Subjects for biological sample collection were drawn from four representative field corrals, with *n* = 4 monkeys selected per corral. These monkeys, within each corral, and as a whole, exhibited non-social scores that were distributed evenly across the frequency of non-social behavior (i.e., the mean and SD of non-social behavior frequency did not differ by corral), thereby enabling analysis of which biological measures are associated with female monkey sociality.

### Biological sample collection and processing procedures

Samples were collected between 0830 and 1100 to minimize any potential circadian effects on the biological measurements. Collection of both CSF and blood samples was accomplished within 10–15 min of initial corral entry. Four monkeys were sampled per day, with two monkeys per corral captured simultaneously. Briefly, each subject was captured from her home corral, rapidly immobilized with ketamine (0.015–0.075 mg/kg), and moved to an indoor procedure room and administered dexmedetomidine (0.015–0.075 mg/kg) to manage pain and sedation. Immediately following relocation, CSF (2 mL) was drawn from the cisterna magna using standard sterile procedure. CSF samples were immediately aliquoted into 1.5 mL siliconized polypropylene tubes and flash-frozen on dry ice.

Next, whole blood samples were drawn from the femoral vein. Twelve mL of whole blood was collected into chilled Ethylenediaminetetraacetic acid-treated vacutainer tubes and immediately placed on wet ice. These samples were then promptly centrifuged (1600×*g* at 4 °C for 15 min), the plasma fraction aliquoted into 1.5 mL polypropylene tubes, and flash-frozen on dry ice. All biological samples were stored at − 80 °C until quantification.

After sample collection, each subject was prophylactically administered metoclopramide (0.2–0.5 mg/kg, an anti-emetic) and ketoprofen (up to 5 mg/kg, a nonsteroidal anti-inflammatory drug), as per veterinary recommendation, to relieve nausea and any pain when waking up from the anesthetic. Additionally, replacement fluids were given if needed per veterinary guidelines. Subjects were placed in a standard laboratory cage located in a hospital/transition room for recovery overnight, and then returned to their home corrals the next day.

### CSF and plasma neuropeptide quantifications

CSF and plasma OXT and AVP concentrations were quantified using commercially available enzyme immunoassay kits (Enzo Life Sciences, Farmingdale, NY) [2, 17–20, 46]. These kits have been validated for use in rhesus monkeys and are highly specific and exclusively recognize OXT and AVP, respectively, and not related peptides (i.e., the OXT cross-reactivity with AVP is < 0.02%; and the AVP cross-reactivity with OXT is < 0.001%). A research scientist blinded to experimental conditions performed sample preparation and OXT and AVP quantification following established procedures [2, 17]. Briefly, plasma samples (1000 μL per animal) for each peptide were extracted per manufacturer's instructions to preclude known matrix interference effects of large blood-borne proteins in the accurate quantification of the neuropeptides [47] and evaporated using compressed nitrogen. Each evaporated plasma sample was reconstituted in 250 μL of assay buffer prior to OXT and AVP quantification to provide sufficient sample volume to run each sample in duplicate wells. This practice ensured that the plated samples contained high enough peptide quantities to be read above the limit of detection (15 pg/mL for OXT and 2.84 pg/mL for AVP). CSF samples were directly assayed (without prior extraction) for OXT and AVP. All CSF and plasma samples were assayed in duplicate (100 µL per well) with a tunable microplate reader (Molecular Devices, CA) for 96-well format per manufacturer’s instructions. The intra-assay and inter-assay coefficients of variation were below 10% for both OXT and AVP.

### Statistical analyses

Data were analyzed using JMP Pro 16 (SAS Institute Inc., Cary, NC). We first used a General Linear Model (GLM) to test whether non-social behavior frequency predicted mSRS-R score in female rhesus monkeys, as previously found for male rhesus monkeys [10]. We included age, the number of females in the corral, and the number of kin in the corral as control variables. As noted above, because rhesus monkeys are a matrilineal species, we also included dominance rank as a predictor. We initially tested for an interaction between non-social behavior frequency and dominance rank (i.e., to assess whether any effect of non-social behavior frequency on mSRS-R score was dependent upon dominance rank). As this interaction was not significant (see below), we removed this term from the final model following best practice [48]. Proportion of observations spent with the mother was also initially included as a control variable. However, in doing so, *n* = 8 subjects with no mother in the corral were excluded due to missing data for this variable. As this control variable was nonsignificant, it was removed from the final analysis to maximize sample size. The final model included data from *n* = 87 animals. (One subject was excluded due to missing data, i.e., the number of kin in its corral.) The assumptions of GLM (homogeneity of variance, normality of error, and linearity) were confirmed post-hoc. No transformations were required. We next performed secondary analyses (using the same statistical model) to test whether any of the social behavior frequencies could predict mSRS-R score. Given the secondary nature of these analyses, we adopted a Bonferroni-corrected critical alpha of 0.01 for the social behavior measures.

We next used GLM to test whether blood concentration of AVP predicted CSF AVP concentration, to assess the feasibility of using blood AVP concentration as a proxy measure for brain-related behavioral processes. We included age, the number of females in the corral, and the number of kin in the corral as control variables. Dominance rank and the blood AVP concentration were included as predictors. Blood AVP concentration was (natural)-log-transformed to meet the linearity assumption for GLM. This analysis included 14 animals (2 of the original 16 animals were excluded for missing AVP data). An identical analysis was run for OXT, except that blood OXT concentration was not log-transformed, and 15 animals were included (only 1 of 16 was excluded due to missing OXT data). No other transformations were required for either analysis.

We then used a GLM to test whether our biological measurements predicted non-social frequency, or (in a separate analysis), mSRS-R score. We included age, the number of females in the corral, and the number of kin, as control variables. Dominance rank, CSF OXT concentration, and CSF AVP concentration were included as predictors. These analyses included 14 subjects (2 of the 16 subjects sampled were excluded due to missing neurochemical data). No transformations were required.

Finally, we used a similar model to test whether CSF OXT and CSF AVP concentrations predicted dominance rank (in the same analysis). This GLM included age, the number of females in the corral, and the number of kin, as control variables. This analysis included 14 animals (2 of the 16 subjects sampled were excluded due to missing neurochemical data). No transformations were required.

## Results

**Fig. 1:**
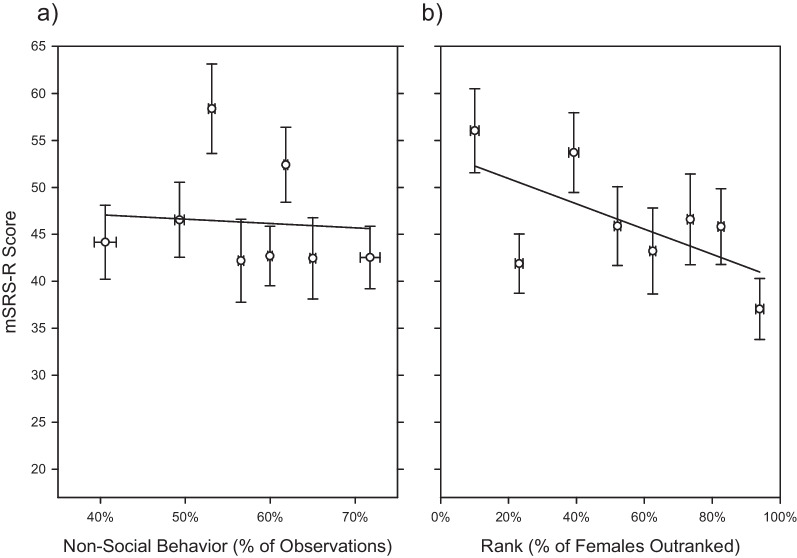
Predictors of mSRS-R score in *N* = 87 female rhesus macaques. **a** Non-social behavior frequency did not significantly predict mSRS-R score. **b** Dominance rank significantly predicted mSRS-R score, with higher ranking individuals having lower autistic-like trait burden. The least-squares-line is plotted. The mSRS-R score was corrected for each data point for the other factors in the analysis, equivalent to figuring a Least squares mean (LSM) and SE for a categorical variable. Then, for ease of visualization, the data were binned into eight quantiles, and the mean and SE plotted. The continuous nature of both non-social behavior frequency and mSRS-R scores are visualized

Non-social behavior frequency and mSRS-R scores were continuously distributed across the general female rhesus monkey population, as we had previously observed for male rhesus monkeys [10]. However, unlike males, non-social behavior frequency did not significantly predict mSRS-R score in females (*F*_1,81_ = 0.0787; *P* = 0.7789; Fig. 1a). In contrast, dominance rank did significantly predict mSRS-R score (*F*_1,81_ = 5.5181; *P* = 0.0213; Fig. 1b), which we have not previously observed in male rhesus monkeys [10, 12, 49]. As noted above, the initial model tested for an interaction between non-social behavioral frequency and rank, which was non-significant (*F*_1,80_ = 0.0007; *P* = 0.9795), and therefore removed from the final model. In our secondary follow-up analyses, none of the social behavior measures predicted mSRS-R score (*P* < 0.05 for all, critical alpha = 0.01).

**Fig. 2:**
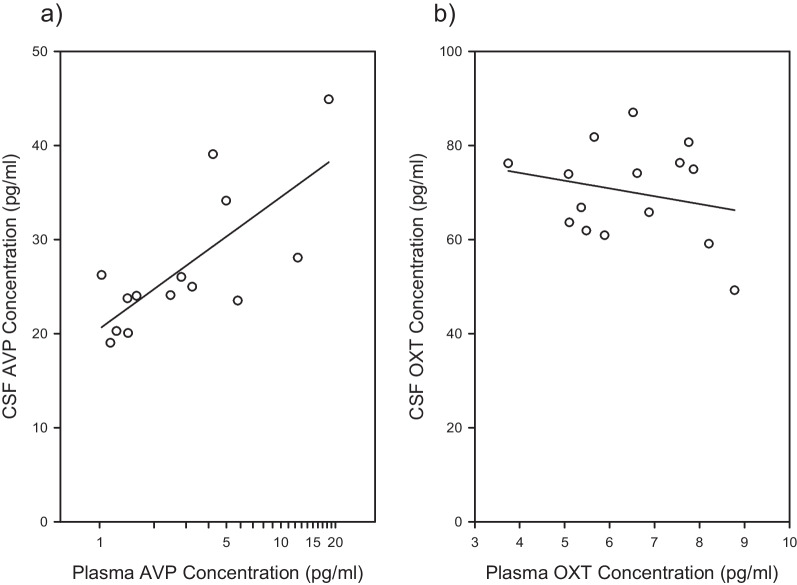
Plasma neuropeptide concentration prediction of CSF neuropeptide concentration. **a** Plasma AVP concentration significantly predicts CSF AVP concentration (*n* = 14). **b** In contrast, plasma OXT concentration does not significantly predict CSF OXT concentration (*n* = 15) in the same animals, using blood plasma and CSF aliquots from the same sample collection. The least-squares-line is plotted. CSF concentration of each neuropeptide was corrected for each data point for the other factors in the analysis, equivalent to figuring a LSM and SE for a categorical variable

Blood AVP concentration significantly and positively predicted CSF AVP concentration (*F*_1,8_ = 6.1523; *P* = 0.0381; *r* = 0.659; Fig. 2a). In contrast, blood OXT concentration did not significantly predict CSF OXT concentration (*F*_1,9_ = 0.2745; *P* = 0.6130; *r* = − 0.1716; Fig. 2b). Importantly, the significant relationship between blood AVP concentration and CSF AVP concentration indicates that these analyses had sufficient power.

In our representative sample subset, we observed no relationship between CSF OXT concentration (*F*_1,7_ = 0.7429; *P* = 0.4173) or CSF AVP concentration (*F*_1,7_ = 1.660; *P* = 0.2386) and non-social behavior frequency. Similarly, neither CSF AVP concentration (*F*_1,7_ = 0.1730; *P* = 0.6900) nor CSF OXT concentration (*F*_1,7_ = 3.6629; *P* = 0.0972) predicted mSRS-R score. However, even in this smaller sample subset, dominance rank continued to predict mSRS-R score, such that higher dominance rank was predictive of a lower mSRS-R score (F_1,7_ = 11.04; *P* = 0.0127). Importantly, this finding indicates that despite the small sample size, there was sufficient power to detect effects seen in the larger data set.

Finally, neither CSF AVP concentration (*F*_1,8_ = 0.1690; *P* = 0.6918) nor CSF OXT concentration (*F*_1,8_ = 2.5659; *P* = 0.1479) significantly predicted dominance rank.

## Discussion

Here, we observed a continuous distribution of non-social behavior frequency and mSRS-R scores in females from the general CNPRC rhesus monkey population, similar to prior findings from male rhesus monkeys in this colony [10]. However, notable sex differences emerged in our subsequent analyses: Social and non-social behavior frequency measures were unrelated to mSRS-R scores in females (unlike in males), and we found no relationships between any neuropeptide measure and social functioning in females (in contrast to prior CSF AVP findings in male rhesus monkeys and male and female autistic children). Instead, we found that dominance rank significantly predicted autistic-like trait burden in female rhesus monkeys, such that higher-ranking individuals exhibited fewer autistic-like traits compared to lower-ranking ones.

What does one make of this puzzling pattern of results? Although we did assess younger female monkeys here, and autistic traits tend to be detected later in life more frequently in women than in men [26, 50], our prior rigorous psychometric assessment of the mSRS-R instrument in both male and female monkeys across a large age range does not support age as a likely determinant of the failure to detect relationships between social behavior frequencies and mSRS-R scores here [10]. The measured range of female mSRS-R scores in this sample is also similar to previous work [10]. Another possibility is that the mSRS-R tool lacks sensitivity in capturing how female monkeys express autistic-like traits; existence of a distinct manifestation of autistic strengths and difficulties is thought to be specific to females (i.e., the Female Autism Phenotype) [30, 51, 52]. However, for this to be the case here, the human SRS instrument and original 36-item mSRS instrument would need to have been developed and tested using only male human and rhesus monkey subjects. Yet, the SRS has been tested extensively in female humans [53–55], and the mSRS instrument was created using a nearly all-female rhesus monkey sample (*N* = 105 monkeys, *n* = 91 females) [43]. Intriguingly, a similar significant effect of rank on mSRS score was reported in this original 36-item mSRS instrument [43]. This instrument was also developed in a different primate colony, by an independent investigative team, serving to reinforce our thinking below.

Although disappointing from an animal model development perspective, the most likely interpretation of the present findings is that the strong matrilineal social organizational system of this species may limit the usefulness of low sociality in female rhesus monkeys as a tractable model for ASD. Social interactions within a rhesus monkey troop are largely governed by the social standing of maternal kin. Female and male offspring both inherit their mother’s dominance rank [36, 56–58], with high- and low-ranking animals experiencing the social landscape appreciably differently from birth [59–61]. Females remain in their natal groups for life, and their dominance rank remains stable across lifespan development [62, 63]. Males, however, relinquish their mother’s rank when they reach puberty and emigrate to a new social group. Upon their arrival, immigrant males typically occupy the lowest position in the social hierarchy, and those who ultimately attain greater dominance rank typically do so through social skill and cunning [64, 65]. Given the fixed versus fluid experience of dominance rank on females’ and males’ social interactions, respectively, and the different selective pressures faced by females and males of this species, it is possible that the mSRS-R score in female rhesus monkeys is essentially a proxy for dominance rank (or at least overlaps with it substantially), whereas the mSRS-R score in male rhesus monkeys instead reflects the underlying and heritable constructs of quantitative variation in social functioning and social abilities, as reported for male rhesus monkeys previously [10, 12, 49].

Although one might have expected that CSF OXT concentration would be related to measures of female social functioning, we have not previously found this to be the case in a sample of female children with and without ASD [18]. As noted above, it was CSF AVP concentration that was found to be a robust predictor of group differences in sociality (i.e., in low- versus high-social male rhesus monkeys; in male and female autistic children versus children without ASD), as well as individual differences in social functioning, including autistic-like trait burden and social symptom severity (in male rhesus monkeys and in male children with ASD, respectively) [2, 17, 18]. In contrast to these findings, CSF AVP concentration in female rhesus monkeys was unrelated to any behavioral measure (i.e., social and non-social behavior frequencies; mSRS-R scores; dominance rank). These collective findings reinforce the possibility that the construct of low sociality in female rhesus monkeys may provide only limited insight into the biological basis of ASD.

The one significant neurochemical result we found in this study was that young female monkeys showed a positive association between blood AVP and CSF AVP concentrations. This finding is in agreement with prior research documenting a significant relationship between blood and CSF AVP concentrations in two human newborn cohorts, one undergoing clinical sepsis evaluation (all were sepsis negative) [66], and one with hypoxic-ischemic encephalopathy [67]. Although there is evidence that blood and CSF AVP concentrations may continue to be somewhat correlated in childhood [68], blood and CSF AVP concentrations no longer appear to be linked in adulthood [69, 70], a notion also corroborated by findings from our male monkey model [2]. We have previously argued that these discrepant findings in the relationship between blood and CSF AVP concentrations may be due to significant developmental changes in the anatomy of the blood–brain barrier [5], whereby the endothelial junctions of the brain’s venous system are not as tightly formed early in life, potentially allowing for larger molecules (including neuropeptides) to flow more freely between the brain and body [71]. Although of less relevance here, these findings support the notion that studies of neuropeptide biology in young human and non-human primates may be able to use blood, a far more accessible fluid than CSF, as a proxy measure for brain-related behavioral processes. Why we observed a correlation between AVP concentrations in concomitantly collected blood and CSF samples, but not a similar positive correlation in OXT concentrations (from the same animals and sample aliquots), remains unclear.

### Limitations

This research had limitations which require discussion. First, despite using a model organism that possesses the complex cognitive and social abilities required to model ASD, we recognize that all animal models serve as approximations for human phenomena, including neurodevelopmental disorders. Second, female humans show lower phenotypic expression of genetic susceptibility in the general population [72], and can manifest ASD symptoms substantially differently from males [30, 51, 52]. Careful genetic screening of ASD susceptibility genes combined with deep phenotyping (including social network analysis) may be a path forward in developing a construct valid model of ASD in female rhesus monkeys, the feasibility of which is beginning to be investigated [3]. Third, there are various versions of the mSRS instrument [10, 43, 73], and whether use of one modified for younger animals would have yielded different results remains unknown. Of the measures we did employ, greater granularity in subjects’ social interactions (e.g., Did they initiate or merely passively receive social overtures? With whom were they interacting—maternal kin or unrelated peers?), may have yielded data more consistent with their mSRS-R scores. Fourth, due to budgetary constraints, we were unable to compare female and male monkeys directly here. However, we employ standardized behavioral collection, biological sampling, and neurochemical quantification protocols across all of our studies, thereby minimizing the impact of variability in experimental procedures on our findings. Fifth, our neurochemical measures were restricted to AVP and OXT in this proof-of-concept study. Future investigations of female (and male) social functioning should consider expanded biomarker discovery efforts, including targeted proteomic, and hypothesis-independent mass spectrometry, approaches.

## Conclusions

In summary, this study determined that non-social behavior frequency and autistic-like traits were continuously distributed across the general female rhesus monkey population, as previously found for male rhesus monkeys in this colony. Unlike in males, however, social and non-social behavior frequencies were unrelated to autistic-like trait burden in females. Additionally, dominance rank was the most significant predictor of autistic-like traits in female monkeys (a finding not previously observed in male monkeys), and CSF neuropeptide concentrations were unrelated to any measure of female social functioning (in contrast to prior CSF AVP findings in male rhesus monkeys and male and female autistic children). Results from this study underscore the complex interplay between species-typical social behavior, autistic-like traits, dominance rank, and neurochemical profiles, potentially reflecting unique mechanisms involved in the expression of social interactions and autistic-like traits between sexes. Nevertheless, from an animal model development perspective, this preliminary evidence suggests that the strong matrilineal organization of this species may limit the usefulness of low sociality in female rhesus monkeys as a tractable model for ASD.

## Data Availability

The datasets used and/or analyzed during the current study are available from the corresponding author on reasonable request.

## Abbreviations

AAALAC International: Association for Assessment and Accreditation of Laboratory Animal Care International
ASD: Autism spectrum disorder
AVP: Arginine vasopressin
CNPRC: California National Primate Research Center
CSF: Cerebrospinal fluid
EDTA: Ethylenediaminetetraacetic acid
GLM: General Linear Model
IACUCs: Institutional Animal Care and Use Committees
LSM: Least squares mean
mSRS: macaque Social Responsiveness Scale
mSRS-R: macaque Social Responsivness Scale-Revised
OXT: Oxytocin
SD: Standard deviation
SE: Standard error
SRS: Social Responsiveness Scale
